# Surgical Antimicrobial Prophylaxis: Clinical and Economic Impacts on Surgical Site Infection Prevention and Healthcare System Sustainability

**DOI:** 10.3390/idr18050109

**Published:** 2026-09-27

**Authors:** Marta Francisca de Fátima Fragoso, Jorge Rufino Ribas Timi, Aline da Silva Paula, Leticia Ramos Dantas, Felipe Francisco Tuon

**Affiliations:** 1 Department of Surgery, Universidade Federal do Paraná (UFPR), Curitiba 80060-270, PR, Brazil; marta.fragoso@ebserh.gov.br (M.F.d.F.F.); jorgetimi@terra.com.br (J.R.R.T.); silvapaula@ufpr.br (A.d.S.P.); 2 Laboratory of Emerging Infectious Diseases, School of Medicine, Pontifícia Universidade Católica do Paraná, Curitiba 80215-901, PR, Brazil; leticia.dantas@pucpr.edu.br

**Keywords:** surgical antimicrobial prophylaxis, surgical site infection, pharmacoeconomics, antimicrobial stewardship, value-based healthcare

## Abstract

Background: Surgical antimicrobial prophylaxis (SAP) is a cornerstone of surgical site infection (SSI) prevention and represents one of the most cost-effective interventions in perioperative care. However, inappropriate antimicrobial selection, timing, dosing, and duration remain common, compromising clinical outcomes and increasing healthcare costs. This narrative review summarizes the clinical and economic impacts of SAP and its role in antimicrobial stewardship, value-based healthcare, and healthcare sustainability. Methods: A narrative review was conducted to synthesize current evidence on the clinical and economic impacts of SAP. The literature addressing the burden of SSIs, the clinical and economic impacts of prophylactic strategies, antimicrobial selection and duration, stewardship interventions, and future perspectives for optimizing perioperative infection prevention was reviewed. Results: Available evidence indicates that appropriate SAP reduces SSI-related morbidity and healthcare resource utilization, whereas inappropriate prophylaxis and prolonged antimicrobial exposure increase costs without improving outcomes. In a Brazilian microcosting study, deep sternal wound infection increased hospitalization costs by 152%, and mediastinitis by 188%, compared with uninfected patients. In orthopedic surgery, actual prophylaxis costs were approximately 11-fold higher than guideline-based regimens when inappropriate prescribing predominated. In neurosurgery, prolonged prophylaxis increased antimicrobial costs from US$3.35 to US$20.41 per patient, despite similar SSI rates (3.5% vs. 3.6%). Stewardship interventions have also demonstrated substantial economic benefits; one arthroplasty program improved optimal prophylactic antimicrobial selection from 64.9% to 95.4% and generated estimated savings of US$197,050 per 1000 procedures. Conclusions: Appropriate SAP is a high-value intervention that improves patient outcomes while reducing healthcare costs. Optimizing antimicrobial selection, timing, dosing, and duration through stewardship initiatives is essential to maximize clinical effectiveness, minimize unnecessary antimicrobial exposure, and promote sustainable healthcare systems. Future studies should incorporate standardized pharmacoeconomic methodologies to strengthen evidence-based decision-making and value-based surgical care.

## 1. Introduction

Surgical site infections (SSIs) remain among the most common healthcare-associated infections worldwide and are associated with substantial clinical and economic consequences [1]. Despite advances in surgical techniques, perioperative care, and infection prevention measures, SSIs continue to contribute significantly to postoperative morbidity, prolonged hospitalization, readmissions, reoperations, and increased mortality [2]. Beyond their impact on patient outcomes, these infections impose a considerable financial burden on healthcare systems through increased resource utilization and higher treatment costs [3].

Surgical antimicrobial prophylaxis (SAP) is an established component of perioperative infection prevention and is recommended across a wide range of surgical procedures [4]. However, its clinical and economic value depends on several factors, including appropriate antimicrobial selection, timing of administration, dosing, duration, local epidemiology, patient-specific risk factors, and adherence to evidence-based recommendations. Inappropriate or prolonged prophylaxis may increase antimicrobial exposure and healthcare costs without providing additional protection against SSI.

From an economic perspective, the potential value of SAP derives from the balance between the relatively low cost of preventive antimicrobial therapy and the substantially higher costs associated with postoperative infection and its complications. Nevertheless, the magnitude of this benefit may vary across surgical settings, patient populations, healthcare systems, and implementation strategies. Differences in local antimicrobial costs, SSI incidence, clinical practices, and methodologies used for economic evaluation may also limit direct comparisons among studies [5].

In this context, pharmacoeconomic provides a valuable framework for evaluating the relationship between the costs and outcomes associated with healthcare interventions [6]. Through methodologies such as cost-effectiveness, cost–benefit, cost-minimization, and cost-utility analyses, pharmacoeconomic evaluations support decision-making processes by identifying interventions capable of generating the greatest health benefits while optimizing resource allocation [7]. The application of pharmacoeconomic principles to surgical antimicrobial prophylaxis is particularly relevant because relatively low-cost preventive interventions may avoid costly postoperative complications and improve overall healthcare value [8].

More recently, the growing adoption of value-based healthcare models has reinforced the importance of assessing both clinical outcomes and economic performance when implementing infection prevention strategies [9]. In this scenario, surgical antimicrobial prophylaxis represents a paradigmatic example of an intervention capable of simultaneously improving patient safety, reducing healthcare expenditures, and promoting sustainability within healthcare systems [10].

Therefore, this narrative review aims to summarize the current evidence regarding the clinical and economic impacts of surgical antimicrobial prophylaxis, focusing on surgical site infection prevention, healthcare resource utilization and costs, antimicrobial stewardship initiatives, and healthcare system sustainability.

## 2. Economic Burden of Surgical Site Infections

This narrative review was conducted to provide an updated overview of the clinical and pharmacoeconomic evidence regarding surgical antimicrobial prophylaxis. Relevant literature was identified through searches of [PubMed/MEDLINE, Scopus, Web of Science—insert only the databases actually used], using combinations of terms related to “surgical antimicrobial prophylaxis,” “surgical site infection,” “cost-effectiveness,” “pharmacoeconomics,” “healthcare costs,” “antimicrobial stewardship,” and “value-based healthcare.” Original studies, systematic reviews, meta-analyses, guidelines, and other relevant publications addressing the clinical or economic impact of surgical antimicrobial prophylaxis were considered. Studies were selected according to their relevance to the objectives of this narrative review. Because this was not designed as a systematic review, no formal PRISMA-based study selection process or quantitative evidence synthesis was performed.

Surgical site infections represent one of the most frequent healthcare-associated infections and remain a major challenge for healthcare systems worldwide. For surgical patients, SSIs constitute most nosocomial infections and account for approximately 20% of all hospital-acquired infections [11]. Despite advances in perioperative care and infection prevention strategies, SSIs continue to be associated with significant morbidity, mortality, and healthcare expenditures. According to reports, SSIs account for almost one-third of postoperative fatalities globally, and they annually endanger the lives of millions of people and fuel the development of antibiotic resistance [12]. Their impact extends beyond clinical outcomes, generating substantial economic consequences for patients, healthcare institutions, and society.

The economic burden of SSIs can be categorized into direct, indirect, and intangible costs. Direct costs include expenditures related to additional antimicrobial therapy, diagnostic tests, surgical reinterventions, intensive care unit admissions, prolonged hospitalization, wound care management, and outpatient follow-up. Several studies have demonstrated that patients who develop SSIs require significantly greater healthcare resource utilization compared with non-infected surgical patients, resulting in considerable increases in treatment costs [13].

Among the most important contributors to the economic burden of SSIs is prolonged hospital length of stay. Badia et al. assessed the impact of SSI on health cost in 26 publications from Europe, but the major issue was the homogeneity of data for cost evaluation [14]. Studies have demonstrated that patients who develop SSIs may require an additional 7 to 10 days of hospital stay compared with uninfected surgical patients, largely due to the need for further diagnostic investigations, intravenous antimicrobial therapy, surgical reinterventions, and management of infection-related complications [15]. Moreover, the attributable cost of a single SSI has been estimated to range from approximately US$11,000 to over US$20,000 per case, depending on the type and severity of the infection [16]. These findings highlight the significant economic burden imposed by SSIs on healthcare systems and reinforce the importance of effective preventive strategies aimed at reducing postoperative infectious complications.

In addition to direct medical costs, SSIs generate important indirect costs associated with productivity losses, delayed return to work, long-term disability, and caregiver burden [17]. These consequences may persist long after hospital discharge and substantially increase the overall societal impact of postoperative infections. Furthermore, intangible costs, including pain, psychological distress, reduced quality of life, and decreased patient satisfaction, represent significant but often difficult-to-measure components of the total burden [1].

Several economic analyses have highlighted the financial consequences of SSIs across different surgical specialties. In orthopedic trauma, for example, infection following tibial fracture fixation has been shown to significantly increase resource utilization and overall treatment expenditures, resulting in treatment costs that can be up to 6.5-times higher than uninfected cases [18]. Original economic studies further illustrate the magnitude of this burden. In a Brazilian microcosting analysis of patients undergoing coronary artery bypass grafting, Orlandi et al. compared patients with and without deep sternal wound infection using propensity-score matching. Mean hospitalization costs increased from US$6863 in patients without infection to US$17,329 in those with deep sternal wound infection and US$19,805 in patients with mediastinitis, corresponding to increases of 152% and 188%, respectively. Postoperative length of stay increased from 9.5 days in controls to 37 days in patients with deep infection and 59 days in those with mediastinitis. Hospital stay was identified as the main cost driver [19].

Given the substantial clinical and economic consequences of SSIs, preventive interventions have become a priority for healthcare organizations worldwide. Effective prevention strategies not only improve patient outcomes but also contribute to more efficient resource utilization and greater sustainability of healthcare systems [20]. Within this context, surgical antimicrobial prophylaxis has emerged as one of the most cost-effective interventions for reducing the burden of postoperative infections and optimizing healthcare expenditures.

It should be noted that the economic estimates reported across the included studies were generated in different years, countries, currencies, and healthcare systems. Therefore, direct comparison of absolute monetary values should be interpreted with caution, as differences in inflation, exchange rates, purchasing power, healthcare pricing, and costing methodologies may substantially affect reported estimates. Whenever possible, future pharmacoeconomic studies should report the price year and consider inflation adjustment and purchasing power parity to improve comparability across settings and over time.

## 3. Clinical and Economic Impacts of Surgical Antimicrobial Prophylaxis

Surgical antimicrobial prophylaxis is widely recognized as one of the most cost-effective interventions for preventing SSIs [21]. By reducing the incidence of postoperative infections, SAP not only improves patient outcomes but also decreases healthcare costs estimated at $4.5–10 billion annually in the United States [22]. The relatively low acquisition cost of prophylactic antibiotics, when compared with the high costs associated with the diagnosis and treatment of SSIs, makes SAP an intervention with a highly favorable cost-effectiveness profile confirmed in several studies around the world [23,24]. Even modest reductions in SSI rates may result in significant cost savings [25]. Multiple studies, including a meta-analysis published in 2018 by Allen et al., have demonstrated that the financial costs associated with managing a single SSI often exceed the total costs of prophylactic antibiotic administration for hundreds of surgical procedures [26,27,28].

Evidence from systematic reviews and economic evaluations consistently supports the cost-effectiveness of preoperative antimicrobial prophylaxis across a variety of surgical specialties, with SSI rates ranging from 0 to 71.1% with costs amounting to US$480–22,130 [29]. These studies have shown that appropriate SAP reduces infection rates while simultaneously lowering overall healthcare expenditures. Importantly, the economic benefits are observed not only in high-risk procedures involving implants or prosthetic materials but also in many clean and clean-contaminated surgeries where infection prevention translates into meaningful reductions in postoperative complications [30].

The effectiveness of SAP depends on adherence to evidence-based principles, including appropriate antimicrobial selection, timing of administration, dosage, and duration. Failure to comply with established guidelines may compromise both clinical and economic outcomes. Inappropriate antibiotic choices, delayed administration, unnecessary redosing, and prolonged postoperative prophylaxis increase antimicrobial consumption and healthcare costs without providing additional protection against infection. Consequently, adherence to guideline-recommended practices is essential for maximizing the economic value of prophylactic interventions. A systematic review recovered 859 articles at the databases, with a total of 18 studies selected for synthesis, showing appropriate indication of antibiotic prophylaxis (ranging from 70.3% to 95%), administration of antibiotic at the correct time (ranging from 12.73% to 100%), correct antibiotic choice (ranging from 22% to 95%), adequate discontinuation of antibiotic (ranging from 5.8% to 91.4%), and adequate antibiotic prophylaxis (ranging from 0.3% to 84.5%) [31]. These data demonstrate that we are far from the ideal SAP considering a global panorama. How many SSIs could be avoided yearly, and what about the financial burden?

Several studies have demonstrated that shorter prophylactic regimens, including single-dose strategies, achieve comparable infection prevention outcomes to prolonged antibiotic courses in selected surgical procedures [32]. From a pharmacoeconomic perspective, reducing unnecessary antimicrobial exposure decreases medication expenditures, minimizes adverse drug events, lowers the risk of antimicrobial resistance, and reduces costs associated with monitoring and treatment-related complications [8]. These findings reinforce the principle that greater antibiotic use does not necessarily translate into greater clinical benefit (Table 1).

In addition to reducing direct healthcare costs, SAP contributes to broader economic benefits by improving surgical outcomes, reducing readmissions, accelerating patient recovery, and facilitating earlier return to normal activities [60]. Such benefits are particularly relevant in modern healthcare systems increasingly focused on value-based care, where interventions are evaluated according to their ability to improve outcomes while optimizing resource utilization [61].

## 4. Economic Impact of Appropriate Antimicrobial Selection and Duration

The selection of an appropriate antimicrobial agent should be guided by the most likely pathogens associated with the surgical procedure, local epidemiological patterns, patient-specific risk factors, and antimicrobial stewardship principles [62,63]. In most clean and clean-contaminated procedures, first-generation cephalosporins, particularly cefazolin, remain the preferred agents due to their efficacy against common skin flora, favorable safety profile, narrow antimicrobial spectrum, and low acquisition cost. The use of broader-spectrum antibiotics when not clinically indicated may increase drug expenditures, promote antimicrobial resistance, and expose patients to unnecessary adverse events.

Comparative studies have demonstrated that broader-spectrum agents do not necessarily provide superior protection against SSIs. In some surgical settings, first-generation cephalosporins have shown equivalent or even superior outcomes compared with third-generation cephalosporins while maintaining lower treatment costs (Figure 1). These findings reinforce the importance of evidence-based antimicrobial selection as a strategy to optimize both clinical outcomes and healthcare resource utilization [64].

The duration of prophylaxis represents another critical determinant of economic efficiency. International guidelines consistently recommend limiting prophylaxis to a single preoperative dose or discontinuing antibiotic administration within 24 h after surgery for most procedures [65]. Despite these recommendations, prolonged postoperative prophylaxis remains common in many institutions, often driven by concerns regarding infection risk rather than scientific evidence. Economic benefits have also been demonstrated for pharmacist-led stewardship interventions. In a retrospective propensity-score-matched study of orthopedic internal fixation procedures, Zhou et al. compared 236 patients in each study group. Pharmacist intervention shortened prophylaxis duration from 2.28 to 1.60 days (*p* < 0.001), reduced mean drug costs from US$706.60 to US$610.57 per patient (*p* = 0.001), and reduced length of stay from 11.14 to 10.43 days (*p* = 0.012). The ratio of drug-cost savings to pharmacist-time costs was 28:1, while the corresponding ratio considering antibiotic costs was 2:1 [66].

Several studies have demonstrated that extending prophylaxis beyond the recommended duration does not significantly reduce SSI rates, even for implant-associated surgeries [67]. In contrast, a recent study from Saudi Arabia showed that prolonged antibiotic exposure increases medication costs, nursing workload, laboratory monitoring requirements, and the risk of antibiotic-related adverse events [68]. Additional consequences include *Clostridioides difficile* infection, drug toxicity, allergic reactions, and the emergence of antimicrobial resistance, all of which contribute to increased healthcare expenditures [69].

From a pharmacoeconomic perspective, unnecessary postoperative antibiotic administration represents a low-value practice because it generates additional costs without corresponding clinical benefits. Economic analyses have shown that adherence to guideline-recommended prophylaxis durations can significantly reduce antimicrobial consumption while maintaining equivalent infection prevention outcomes, even in developing countries [70]. The economic consequences of prolonged prophylaxis were also demonstrated in a prospective study of 822 neurosurgical procedures. Ulu-Kilic et al. compared patients receiving prophylaxis for <24 h with those receiving prolonged prophylaxis. The prophylactic antibiotic cost per patient was US$3.35 with guideline-concordant duration versus US$20.41 with prolonged prophylaxis, while SSI rates remained virtually identical (3.5% vs. 3.6%; *p* > 0.05). Thus, prolonged prophylaxis increased antimicrobial expenditure approximately sixfold without providing additional protection against SSI [70].

In a prospective observational study of 412 patients undergoing open reduction and internal fixation, Kumari et al. found that none of the patients received a completely guideline-concordant prophylactic regimen. The mean direct per-patient cost of the prophylaxis actually administered was INR 4467.2, compared with INR 391.5 for the guideline-recommended regimen, representing an approximately 11-fold increase in expenditure. Prolonged postoperative antimicrobial administration was inappropriate in all patients [8].

The financial implications of inappropriate antimicrobial selection and prolonged prophylaxis extend beyond individual patients [71]. At the institutional level, excessive antimicrobial utilization contributes to higher pharmacy expenditures and increased costs associated with antimicrobial stewardship activities and infection control measures [72]. At the healthcare system level, inappropriate antimicrobial use accelerates the development of antimicrobial resistance, creating long-term economic burdens that affect future treatment options and healthcare sustainability [73,74].

## 5. Antimicrobial Stewardship and Value-Based Care

Antimicrobial stewardship programs (ASPs) have emerged as essential components of modern healthcare systems, aiming to optimize antimicrobial use while improving patient outcomes, minimizing adverse events, reducing antimicrobial resistance, and controlling healthcare expenditures [74,75]. Within the surgical setting, stewardship initiatives play a critical role in ensuring appropriate implementation of SAP, thereby enhancing both clinical effectiveness and economic efficiency. Considering the current AI-based models of applications, the surveillance can be more accurate [74,75,76]. Similarly, implementation of an interprofessional antimicrobial stewardship program in total joint arthroplasty increased optimal preoperative antibiotic selection from 64.9% to 95.4% (*p* < 0.001), while reducing unnecessary antimicrobial exposure. The institutional cost–benefit analysis estimated savings of US$197,050 per 1000 arthroplasty procedures [45].

Common issues include the use of unnecessarily broad-spectrum agents, incorrect timing of administration, inappropriate dosing, and prolonged postoperative prophylaxis. Giamarellou et al. published a recent narrative review of the ASP in hospital setting, including some aspects of SSI prophylaxis [77]. These practices increase antimicrobial consumption and healthcare costs without providing additional clinical benefit.

Numerous studies have demonstrated that stewardship interventions can significantly improve adherence to evidence-based guidelines for surgical prophylaxis [63,78,79,80,81,82,83,84]. Strategies such as prospective audit and feedback [85], clinical decision support systems [86], multidisciplinary protocol development [87], pharmacist-led interventions [88,89], educational programs [90,91], and continuous performance monitoring have been associated with substantial improvements in prescribing practices. As a result, healthcare institutions frequently observe reductions in antimicrobial utilization, lower pharmacy expenditures, and improved compliance with recommended prophylaxis protocols.

The stewardship programs contribute to lower rates of surgical site infections, reduced hospital length of stay, fewer readmissions, and decreased utilization of diagnostic and therapeutic resources [91]. These improvements generate significant cost savings while simultaneously enhancing patient safety and quality of care, which can be crucial in developing countries, where sustainability of the healthcare system is essential [92].

The growing emphasis on Value-Based Healthcare (VBHC) further strengthens the importance of stewardship programs [93,94]. Value-based care focuses on maximizing patient outcomes relative to the costs incurred throughout the continuum of care [95,96]. In this framework, healthcare interventions are assessed not only by their clinical efficacy but also by their ability to generate meaningful health outcomes efficiently and sustainably. Surgical antimicrobial prophylaxis represents an excellent example of a high-value intervention, particularly when implemented within a structured stewardship framework [94].

From a value-based perspective, the success of SAP should not be measured solely by reductions in infection rates but also by its contribution to broader healthcare outcomes, including patient safety, quality of life, resource optimization, and long-term sustainability [97,98]. Stewardship programs facilitate this approach by promoting evidence-based decision-making and ensuring that antimicrobial use aligns with both clinical needs and economic considerations.

In addition, multidisciplinary collaboration is fundamental to the success of stewardship initiatives [99,100]. Surgeons, anesthesiologists, infectious disease specialists, pharmacists, microbiologists, infection prevention teams, and hospital administrators all contribute to the development and implementation of effective prophylaxis protocols. As healthcare systems worldwide face increasing financial pressures, aging populations, and rising antimicrobial resistance rates, the integration of antimicrobial stewardship principles into value-based care models becomes increasingly important. By simultaneously improving clinical outcomes and reducing unnecessary expenditures, stewardship programs support the sustainability of healthcare systems while reinforcing the principles of high-quality, patient-centered care.

Therefore, antimicrobial stewardship should be considered not only an infection prevention strategy but also a key component of value-based healthcare, capable of generating substantial clinical, economic, and societal benefits through the optimization of surgical antimicrobial prophylaxis practices.

## 6. Future Perspectives and Healthcare System Sustainability

The future of SAP extends beyond infection prevention and increasingly encompasses broader goals related to healthcare quality, economic efficiency, and system sustainability. As healthcare systems worldwide face rising costs, increasing surgical volumes, aging populations, and the growing threat of antimicrobial resistance, optimizing preventive strategies has become a strategic priority for policymakers, healthcare institutions, and clinical professionals [101,102].

One of the most promising developments in this field is the incorporation of precision medicine into perioperative infection prevention [103]. Traditional prophylactic protocols are generally based on procedure-specific recommendations and population-level risk estimates. However, advances in genomics, microbiome research, and predictive analytics may enable more individualized approaches to antimicrobial prophylaxis [104]. Future strategies may consider patient-specific characteristics, colonization status, microbiological risk profiles, and local epidemiological patterns to optimize prophylactic interventions while minimizing unnecessary antimicrobial exposure.

Artificial intelligence and machine learning technologies also have the potential to transform perioperative infection prevention [74,75]. Predictive models capable of identifying patients at increased risk for SSIs may support more targeted preventive measures, improve resource allocation, and enhance clinical decision-making [50]. Additionally, AI-driven clinical decision support systems may assist healthcare professionals in selecting the most appropriate antimicrobial agent, dose, timing, and duration based on real-time patient and institutional data [105]. However, the use of AI in SAP is still incipient, focused on infection control and prevention [106,107].

The increasing availability of electronic health records and healthcare analytics platforms provides new opportunities for monitoring compliance with prophylaxis guidelines and evaluating outcomes at both institutional and healthcare system levels [108]. Automated surveillance systems may facilitate the identification of prescribing patterns, benchmark performance indicators, and support continuous quality improvement initiatives [109]. Hartmann et al. showed the impact of inadequate surgical antibiotic prophylaxis on perioperative outcome and length of stay, with improvement using automated surveillance system [109]. These tools may contribute to more efficient antimicrobial stewardship programs and promote evidence-based practice across surgical specialties.

Another important area of future development involves the integration of pharmacoeconomic evaluations into routine healthcare decision-making. While the clinical benefits of SAP are well established, economic analyses remain heterogeneous and are often underrepresented in surgical research. Future studies should incorporate standardized pharmacoeconomic methodologies, including cost-effectiveness, cost-utility, and budget impact analyses, to better quantify the value generated by prophylactic interventions. In addition, economic evaluations should adhere to established reporting frameworks, such as the Consolidated Health Economic Evaluation Reporting Standards (CHEERS), to improve methodological transparency, completeness, comparability, and reproducibility across studies. Such standardization will strengthen the evidence available for policy decisions and resource allocation strategies in increasingly constrained healthcare environments.

## 7. Conclusions

Surgical antimicrobial prophylaxis represents one of the most effective and cost-effective strategies for preventing surgical site infections, reducing postoperative morbidity, mortality, healthcare resource utilization, and associated costs. The evidence reviewed demonstrates that appropriate antimicrobial selection, timing, dosage, and duration are critical for maximizing both clinical and economic outcomes. Furthermore, antimicrobial stewardship programs enhance the value of prophylactic interventions by promoting guideline adherence, reducing unnecessary antimicrobial use, mitigating antimicrobial resistance, and optimizing resource allocation. In the context of increasing healthcare expenditures and growing demands for quality and efficiency, surgical antimicrobial prophylaxis should be regarded not only as a cornerstone of infection prevention but also as a strategic component of value-based healthcare. Future advances in precision medicine, artificial intelligence, and healthcare analytics may further improve the effectiveness and sustainability of prophylactic strategies, reinforcing their role in supporting resilient and economically sustainable healthcare systems.

## Figures and Tables

**Figure 1:**
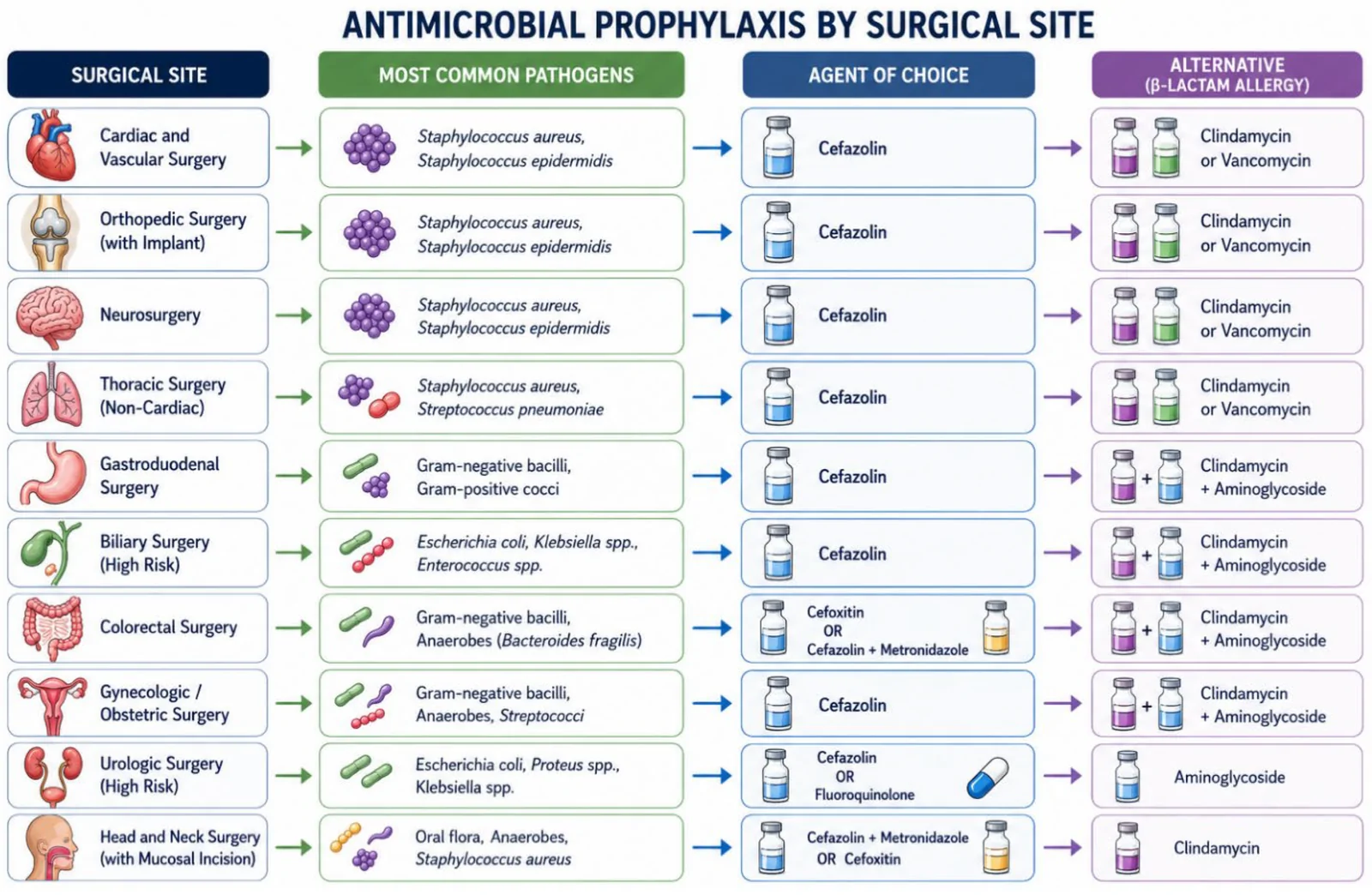
Recommended surgical antimicrobial prophylaxis according to surgical procedure, expected pathogens, and alternative regimens for patients with β-lactam allergy. The figure summarizes the most common microorganisms associated with different surgical sites and the corresponding antimicrobial prophylaxis regimens recommended by international guidelines. Cefazolin remains the preferred agent for most clean surgical procedures due to its efficacy, safety profile, and favorable pharmacoeconomic characteristics. Alternative regimens are presented for patients with β-lactam allergy. Final antimicrobial selection should consider local epidemiology, antimicrobial resistance patterns, institutional protocols, and patient-specific risk factors. The figure was created using Microsoft Powerpoint and ChatGPT-5.

**Table 1:** Overview of key studies evaluating the impact of surgical antimicrobial prophylaxis on surgical site infection prevention, healthcare costs, antimicrobial stewardship, and healthcare system sustainability.

Category	Study	Objective	Main Finding	Design
**Economic Burden of SSI**	Barres-Carsi et al., 2022 [33]	Evaluate economic impact of SSI in tibial fractures	SSI significantly increased healthcare resource utilization and costs	Cohort
	Benenson et al., 2020 [34]	Assess financial impact of healthcare-associated infections	Infections increased hospital resource consumption	Case-control
	Orlandi et al., 2023 [19]	Estimate costs of deep sternal wound infection	SSI generated substantial hospital costs	Economic study
	Nogueira et al., 2024 [35]	Estimate antimicrobial costs associated with SSI	SSI resulted in significant antimicrobial expenditures	Economic study
	Davies et al., 2023 [36]	Model health and economic burden of antimicrobial resistance	Resistance increased projected surgical costs	Methodological study
**Cost-Effectiveness of Surgical Antimicrobial Prophylaxis**	Khan et al., 2022 [37]	Reduce SSI through evidence-based interventions	SSI rate decreased from 30% to 5%	Interventional
	Allen et al., 2018 [26]	Evaluate cost-effectiveness of SAP	SAP reduced SSI and healthcare costs	Systematic review
	Veiga et al., 2025 [38]	Compare single-dose vs. 24 h prophylaxis	Single-dose regimen was sufficient	Randomized trial
	Negri et al., 2024 [39]	Evaluate prophylaxis in hand surgery	Limited reduction in SSI	Systematic review
	Dexter et al., 2023 [40]	Compare multifaceted vs. single interventions	Multifaceted approach reduced SSI by 68%	Prospective cohort
**Antimicrobial Selection and Duration**	Bae et al., 2022 [41]	Compare first- vs. third-generation cephalosporins	First-generation prophylaxis showed lower SSI rates	Retrospective cohort
	Priyanka et al., 2019 [8]	Assess appropriateness and pharmacoeconomics of SAP	Actual costs were 11-fold higher than guideline-based prophylaxis	Prospective observational
	Prebianchi et al., 2023 [42]	Assess type and duration of prophylaxis	Antibiotic selection influenced SSI more than duration	Comparative study
	Oliveira Campos et al., 2024 [43]	Compare antibiotic treatment duration	Longer treatment increased adverse events without reducing recurrence	Comparative study
	Truzzi et al., 2025 [44]	Review prophylaxis recommendations in urology	Evidence supports procedure-specific prophylaxis	Narrative review
**Antimicrobial Stewardship and Value-Based Care**	Hyland et al., 2022 [45]	Evaluate stewardship program in arthroplasty	Savings of US$197,050 per 1000 procedures	Cohort
	Martinez-Sobalvarro et al., 2022 [28]	Assess stewardship effectiveness	Improved adherence and reduced SSI	Systematic review
	Nehad et al., 2022 [46]	Improve compliance with SAP guidelines	Increased adherence to prophylaxis recommendations	Prospective intervention
	Rangchian et al., 2022 [47]	Evaluate pharmacist-led educational intervention	Improved SAP appropriateness and reduced costs	Prospective intervention
	Butt et al., 2019 [48]	Assess pharmacist intervention	Reduced antibiotic use and hospitalization costs	Quasi-experimental
	Bashar et al., 2021 [24]	Evaluate stewardship intervention	Reduced antimicrobial consumption	Cross-sectional study
	Li et al., 2018 [49]	Improve rational SAP prescribing	Multidisciplinary intervention improved compliance	Interventional study
**Risk Factors and Future Perspectives**	Scala et al., 2022 [50]	Identify SSI risk factors using AI	Prophylaxis and surgical specialty predicted SSI	Cohort
	Magalhães et al., 2024 [51]	Identify risk factors in foot and ankle surgery	Comorbidities and operative time increased SSI risk	Cohort
	Batista et al., 2024 [52]	Evaluate SSI in arthroplasty	Age and comorbidities associated with SSI	Observational
	Quiroz-Williams et al., 2024 [53]	Identify risk factors in fractures	Operative time and comorbidities increased risk	Observational
	Gebhardt et al., 2026 [54]	Assess risk factors after CABG	Diabetes and hospitalization time associated with SSI	Retrospective
	Arribas-Leal et al., 2023 [55]	Investigate deep sternal wound infection	Obesity and operative time were risk factors	Observational
	Oliveira et al., 2023 [56]	Assess SSI in neurosurgical and orthopedic procedures	Operative time was a major risk factor	Observational
	Revilla-Pacheco et al., 2024 [57]	Evaluate topical vancomycin	Significant reduction in SSI	Experimental
	Araújo et al., 2023 [58]	Assess preventive measure adherence	Protocol adherence remained suboptimal	Cross-sectional
	Velozo et al., 2024 [59]	Evaluate SAP adherence	Poor adherence associated with higher SSI risk	Observational

## Data Availability

No new data were created or analyzed in this study. Data sharing is not applicable to this article.
